# Approximate Packing of Binary Mixtures of Cylindrical Particles

**DOI:** 10.3390/mi14010036

**Published:** 2022-12-23

**Authors:** Gulfairuz Iniyatova, Assiya Yermukhambetova, Aidana Boribayeva, Boris Golman

**Affiliations:** Department of Chemical and Materials Engineering, School of Engineering and Digital Sciences, Nazarbayev University, Astana 01000, Kazakhstan

**Keywords:** packing, cylindrical particles, binary mixture, microstructure

## Abstract

Particle packing plays an essential role in industry and chemical engineering. In this work, the discrete element method is used to generate the cylindrical particles and densify the binary cylindrical particle mixtures under the poured packing conditions. The influences of the aspect ratio and volume fraction of particles on the packing structure are measured by planar packing fraction. The Voronoi tessellation is used to quantify the porous structure of packing. The cumulative distribution functions of local packing fractions and the probability distributions of the reduced free volume of Voronoi cells are calculated to describe the local packing characteristics of binary mixtures with different volume fractions. As a result, it is observed that particles with larger aspect ratios in the binary mixture tend to orient randomly, and the particles with smaller aspect ratios have a preferentially horizontal orientation. Results also show that the less dense packings are obtained for mixtures with particles of higher aspect ratios and mixtures with a larger fraction of elongated cylindrical particles.

## 1. Introduction

Particle packing is widely used in industry and nature. It is a significant subject that has been studied for a long time. Previous studies of particle packing have primarily focused on the packing of spherical particles due to their simple shape. However, non-spherical particles are usually more involved in industry and everyday life. In chemical engineering, cylindrical particles have been used for heat transfer and catalytic reactions [1,2]. The permeability, catalytic, and heat transfer efficiency of fixed bed reactors are well established to be quantitatively related to the associated packing structure. The complex packing behavior of particles due to the differences in sizes, shapes, density, and other properties still need to be studied thoroughly.

Researchers mainly focused on the study of binary mixtures in order to improve the packing density of non-spherical particles. Previous studies were concentrated on the packing densification of binary spherocylinders [3] and binary cylinders [4]. There is still a need to conduct more work on the packing densification of binary cylindrical particle mixtures.

Recently, the discrete element method (DEM) developed by Cundall and Stark [5] has been increasingly applied to study the packing of non-spherical particles due to its original advantage in providing detailed dynamic information on the individual particle scale. Different approaches can be used to represent and simulate non-spherical particles, such as multisphere (MS) [6] and superquadric (SQ) [7]. The SQ approach provides a general method to model non-spherical particles by describing the particle shape by a mathematical expression and varying five shape parameters [7]. Despite the difficulty of contact detection and force computations, this approach is relatively accurate and has a medium computing cost. A lot of progress has been achieved in the modeling of non-spherical particles utilizing the superquadric particle approach. Soltanbeigi et al. [8] studied the behavior of SQ particles with different blockiness/edge sharpness levels. Zhao et al. [9] focused on how the particle shape affects the calculation speed of the super-ellipsoid DEM. Wang et al. [10] worked on poly-superquadric model based on the superquadric approach. However, this approach still has unresolved problems, such as a complete formulation of the contact algorithm, a detailed formulation of the contact force, a method to stabilize and accelerate the algorithm, the capacity to simulate in complicated geometries, and others. Kodam et al. [11], Guo et al. [12], and Gan and Yu [13] developed the DEM algorithms to simulate the cylindrical particles with planar faces. This representation of cylindrical particles is more precise than that of superquadrics with smoothed boundaries, but it is not yet implemented in the widely used DEM computer programs. Pankratov et al. [14] applied the composed element method to generate complex nonconvex particles by combining the basic convex objects and studied the packing of such particles in a minimal-volume cuboid.

Investigating the behavior of the particle assembly as a whole, as well as the average packing fraction over the domain, would be beneficial [15,16]. However, individual effects of particle morphology, particle physical properties, and particle process parameters on packing characteristics cannot be evaluated using the average packing density. Calculating the packing fraction of the assembly in any specific direction is one way to characterize the variation of packing of the assembly in order to gain an understanding of how the packing differs in that direction. The planar packing fraction on specified planes along the desired direction could be calculated. The planar packing fraction is the ratio of the overall area of the plane to the cumulative area of the geometries imprinted onto it by the particles. A few investigations on the packing fraction for spherical particles have been conducted [17,18]. A recent study by Pola et al. [19] evaluated a voxelization based post-processing workflow to analyze the packing structure of non-spherical particle assembles obtained from DEM simulations of either superquadric or multisphere particles. So far, however, there has been little discussion about such studies on non-spherical particles.

The particle-centered technique, such as the Voronoi tessellation (VT), has been widely used in the design of local cells for spherical packings [20,21,22,23]. Partitioning local cells of irregular packings is complex. Nevertheless, such kind of analysis has been conducted for ellipsoidal packing [24]. For example, Schaller et al. [25] reported a Voronoi analysis of the packing of monosized frictional ellipsoids. They demonstrated that the local packing fraction distribution can be correlated with the overall packing fraction. Schröder-Turk et al. [26] utilized Minkowski tensors to quantify the characteristics of 3D sphere packing in conjunction with Voronoi tessellation. Recently, Zhao et al. [27] explored the universal characteristics in sheared granular materials composed of poly-superellipsoidal particles by using the set Voronoi analysis. In this paper, we use a newly developed approach called set Voronoi tessellation to partition local cells of non-spherical packings [24,28,29,30]. Based on the generalized Voronoi tessellation, the set Voronoi tessellation separates the point clouds on the particle surface inside the package. This results in a sequence of subcells splitting the total packing area, each covering one point on the particle surface. Subcells with the same particle surface points are then joined to produce a Voronoi cell that surrounds the particle [31].

Therefore, the aim of the study is to analyze thoroughly the porous structure of binary mixtures of cylindrical particles of different aspect ratios focusing on the local porosity structure. For this purpose, cylindrical particles are modeled by the SQ approach, packing densification of binary mixtures of the cylindrical particles is simulated using the DEM, the planar packing fraction is calculated by applying voxelization code, and finally, the microstructure of packing is analyzed by VT method for the poured packing of binary mixtures.

The main contribution of this paper is to expand our understanding of the packing structure of binary mixtures of cylindrical particles with various aspect ratios. The authors compare the local packing structure of compacts using the Voronoi tessellation approach and analyze the planar packing fraction distributions along all axes. They also contribute to the study of the orientation of cylindrical particles in the compacts and the spatial distribution of different aspect ratio particles in the compact cross-section.

The remainder of the paper is organized as follows. Section 2 details the methods used in this research. The generation of cylindrical particles using superquadrics, formation of packings of binary cylindrical particle mixtures under the pouring conditions by DEM, calculation of planar packing fraction distributions by voxelization method, and analysis of packing porous structures by Voronoi tessellation are described in this section. The obtained results are presented and discussed in Section 3. Finally, some conclusions are given in Section 4.

## 2. Materials and Methods

The present work follows our previous work on microstructure analysis of compacts of equal-size cylindrical particles by DEM [32]. It focuses on the analysis of the packing structure of binary mixtures of cylindrical particles with different aspect ratios.

### 2.1. Cylindrical Particle Generation

The cylindrical particles were generated using the SQ approach. The particle shape and size are determined by the geometric parameters of the superquadric, such as half-lengths and sharpness indices. The so-called inside-outside function defining the superquadric for a three-dimensional particle has the following form [33]:

(1) $$f\left(x,y,z\right)={\left({\left|\frac{x}{a}\right|}^{n_{2}}+{\left|\frac{y}{b}\right|}^{n_{2}}\right)}^{\frac{n_{1}}{n_{2}}}+{\left|\frac{z}{c}\right|}^{n_{1}}-1=0,$$

where *a*, *b*, and *c* are the superquadric half-lengths along the *x*, *y*, and *z* axes, respectively. The shape sharpness parameter $n_{1}$ describes the shape of the cross-sections in the *y*-*z* and *x*-z planes, and the parameter $n_{2}$ defines the shape of the cross-section in the *x*-*y* plane. The inside-outside function by Equation (1) is specified in superquadric-centered local coordinates. 

The orientation of the particle is described with the help of rotation matrix A, which transforms coordinate vectors in global space to coordinate vectors in the body-fixed frame [34].

(2) $$A=\left(\begin{array}{ccc} 1-2\left(q_{2}^{2}+q_{3}^{2}\right) & 2\left(q_{1}q_{2}-q_{0}q_{3}\right) & 2\left(q_{1}q_{3}+q_{0}q_{2}\right)\\ 2\left(q_{1}q_{2}+q_{0}q_{3}\right) & 1-2\left(q_{1}^{2}+q_{3}^{2}\right) & 2\left(q_{2}q_{3}-q_{0}q_{1}\right)\\ 2\left(q_{1}q_{3}-q_{0}q_{2}\right) & 2\left(q_{2}q_{3}+q_{0}q_{1}\right) & 1-2\left(q_{1}^{2}+q_{2}^{2}\right) \end{array}\right).$$

Here, $q_{0}$ $,\;q_{1}$ $,\;q_{2\;}$ and $q_{3\;}$ are the quaternions given as

(3) $$q_{0}=\cos\left(\frac{\alpha }{2}\right),q_{1}=\hat{x}\cdot \sin\left(\frac{\alpha }{2}\right),q_{2}=\hat{y}\cdot \sin\left(\frac{\alpha }{2}\right),q_{3}=\hat{z}\cdot \sin\left(\frac{\alpha }{2}\right),$$

and $\hat{(x}$, $\hat{y}$, $\hat{z)}$ is the unit vector defining the axis of rotation, and α is the rotation angle around this axis.

The Euler angles ϕ, θ, and ψ can be calculated from quaternions as

(4) $$\left[\begin{array}{c} \phi \\ \theta \\ \psi \end{array}\right]=[\begin{array}{c} \tan^{-1}\left(\frac{2\left(q_{0}q_{1}+q_{2}q_{3}\right)}{1-2\left(q_{1}{}^{2}+q_{2}{}^{2}\right)}\right)\\ \sin^{-1}\left(2\left(q_{0}q_{2}-q_{3}q_{1}\right)\right)\\ \tan^{-1}\left(\frac{2\left(q_{0}q_{3}+q_{1}q_{2}\right)}{1-2\left(q_{2}{}^{2}+q_{3}{}^{2}\right)}\right) \end{array}].$$

The superquadric parameters were chosen to generate cylindrical particles that have the same volumes. Table 1 lists the dimensions of cylindrical particles and the parameters used for their generation.

### 2.2. Discrete Element Method

The Aspherix program by DCS Computing GmbH [35] was used to simulate the packing of cylindrical particle mixtures. The calculation of the particle position and orientation is based on Newton’s second law of motion. The translational and rotational motions of particles are described by the following equations:

(5) $$m_{i}\frac{d^{2}x_{i}}{dt^{2}}=F_{i}\;,$$

(6) $$L_{i}=T_{i},$$

where $m_{i}$ is the *i*th particle mass, $x_{i}$ is the particle position, t is time,$\;F_{i}$ and $T_{i}$ are the sums of forces and torques acting on the *i*th particle, $L_{i}$ is the angular momentum of the particle, $L_{i}=I_{i}\cdot \Omega_{i},\;I_{i}$ is the tensor of inertia, and $\Omega_{i}$ is the angular velocity in the observer-fixed coordinate system.

For irregularly shaped particles, Equation (6) is formulated as

(7) $${\hat{I}}_{i}{\dot{W}}_{i}+W_{i}\cdot {\hat{I}}_{i}W_{i}=A_{i}{}^{-1}T_{i},$$

where ${\hat{I}}_{i}$ is the particle tensor of inertia, $W_{i}$ is the angular velocity, $W_{i}=A_{i}{}^{-1}\Omega_{i}$, and $A_{i}$ is the rotation matrix defined by Equation (2).

The forces and torques affecting the particle are calculated as

(8) $$F_{i}=F_{i,contact}+F_{i,\;gravity}+F_{i,\;external},$$

(9) $$T_{i}=T_{i,contact}+T_{i,external}.$$

The Hertz–Midlin contact model is used in the present study [36].

The mechanical and physical properties of the particles and the DEM simulation parameters are shown in Table 2. Samples of binary mixtures of cylinder particles with different aspect ratios (Table 3) were modeled by placing 12,000 cylinders in a container with the size of 0.064 m × 0.064 m × 0.13 m. In order to simulate the so-called poured packing conditions when the packing is formed by pouring particles into a vessel from the vessel top, a block region was set on the top of the container, and cylindrical particles with random orientation were generated in the container. Then, the bottom block plane was removed, and the particles fell under gravity into the container. This simulation setup closely resembles the poured packing conditions.

### 2.3. Calculation of Planar Packing Fraction

Planar packing fraction is a measure of the packing density of a particle assembly on a certain plane. In order to obtain planar packing fraction by voxelization method, the open-source program [19] was applied to DEM simulation output. The domain was discretized into voxels, and then voxels located inside the particles were identified. The packing fraction on the plane is the ratio of the number of voxels inside the particles to the total number of voxels on the plane.

### 2.4. Voronoi Tessellation

The open-source program PySetVoronoi [29] was used to calculate the Voronoi tessellation and analyze the obtained Voronoi diagram. Output data from DEM simulation were employed in the PySetVoronoi program to recreate compacts of cylindrical particles. Based on the results of Voronoi tesselation, the reduced volume and the reduced free volume of Voronoi cells were calculated as *V_c_*/*V_p_* and *V_c_*/*V_p_*−1, respectively [32]. Here, *V_c_* is the Voronoi cell volume and *V_p_* is the cylindrical particle volume. 

## 3. Results and Discussion

### 3.1. DEM Simulation Results

**Figure 1:**
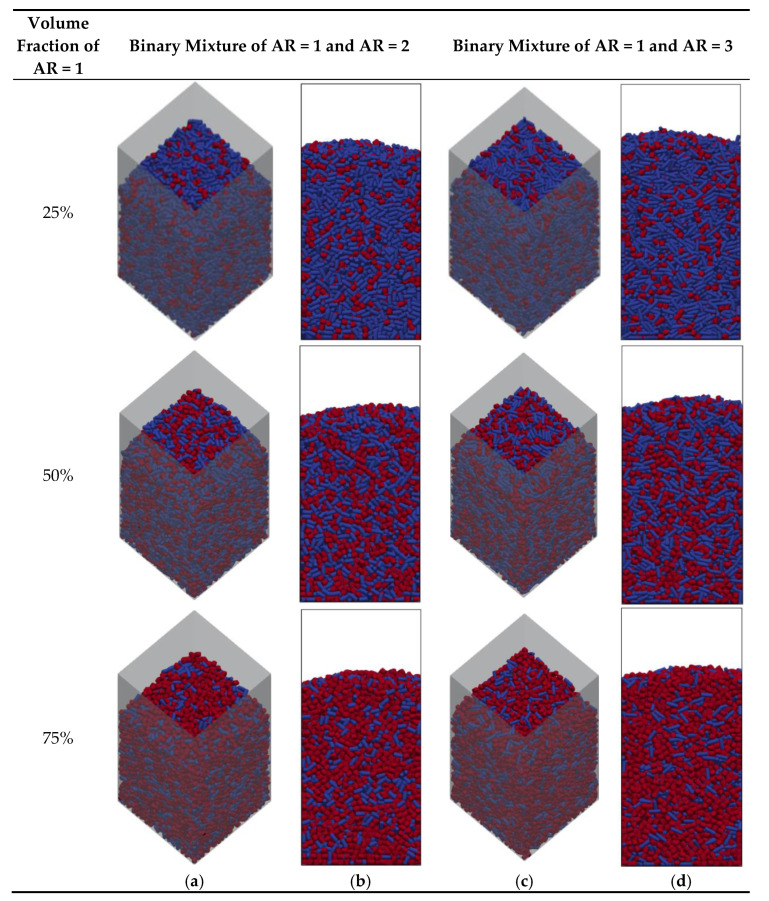
Visualization of binary mixtures of cylinders simulated using DEM: (**a**,**c**) overall bed view and (**b**,**d**) front view (red particles of AR = 1, blue particles of AR = 2 and AR = 3).

**Figure 2:**
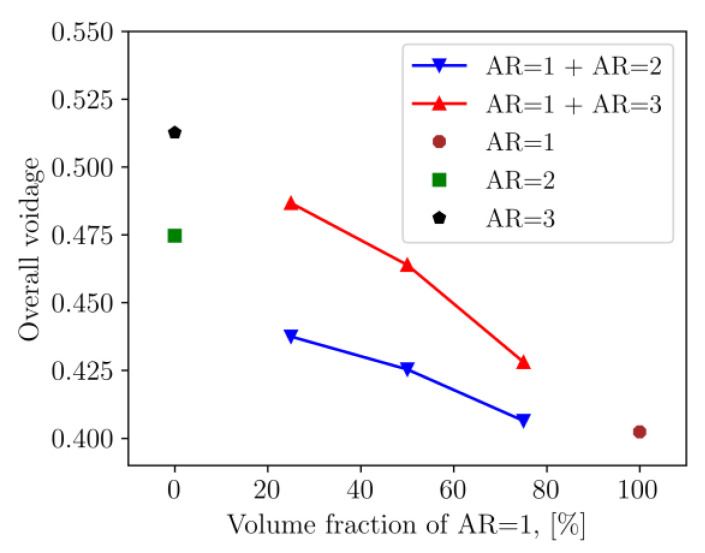
Overall voidage of binary mixture of cylindrical particles.

Figure 1 shows the visualization of the packings of binary cylindrical mixtures with different volume fractions. As seen in Figure 1b, the height of the packing varies depending on the aspect ratio of particles and the composition of the particle mixture. The densest packing of the lowest height was obtained for the mixture of cylindrical particles with close aspect ratios (AR = 1 and AR = 2) and the largest proportion of cylinders with AR = 1. Thus, the elongated particles contribute to the decrease in packing density. To prove that, the overall voidage of packed samples was measured by the voxelization method, and the results are demonstrated in Figure 2 for both monodisperse and binary mixture packings. The packings of the mixtures of cylinders with AR = 1 and AR = 2 are denser than those generated by mixtures of cylinders with AR = 1 and AR = 3 and the overall packing voidage decreases with decreasing volume fraction of elongated particles. These trends are similar to those observed in physical experiments on the packing of wood cylinders of various lengths [38] and simulated experiments on the packing of different aspect ratio cylinders with planar faces [13]. The overall packing voidage is in the range of 0.4–0.52 (Figure 2), which is similar to experimental values reported by Tengri et al. [39] for the pouring packing of acrylic cylindrical particles.

**Figure 3:**
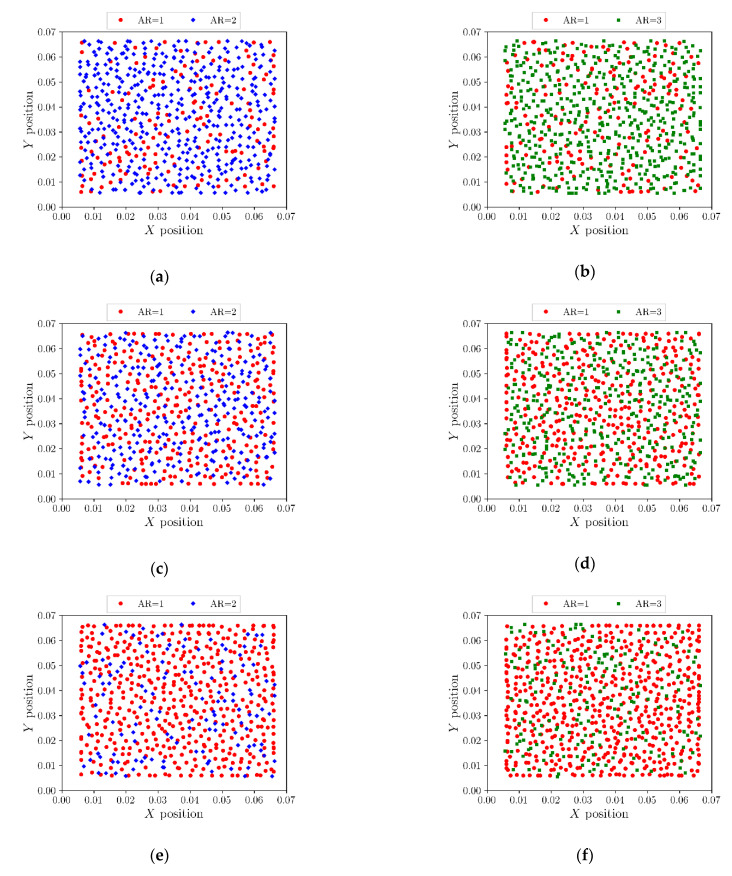
Projected positions of particles in horizontal plane for binary mixtures of (**a**) AR = 1 (25%) and AR = 2 (75%), (**b**) AR = 1 (25%) and AR = 3 (75%), (**c**) AR = 1 (50%) and AR = 2 (50%), (**d**) AR = 1 (50%) and AR = 3 (50%), (**e**) AR = 1 (75%) and AR = 2 (25%) and (**f**) AR = 1 (75%) and AR = 3 (25%).

The projected positions of particles in the horizontal plane are shown in Figure 3. In mixtures, particles with high aspect ratios tend to move further from the wall. This pattern is clearly seen in Figure 3c,d, where even for binary mixtures with equal volume fractions of particles of different aspect ratios, we can observe mainly particles with AR = 1 near the walls.

**Figure 4:**
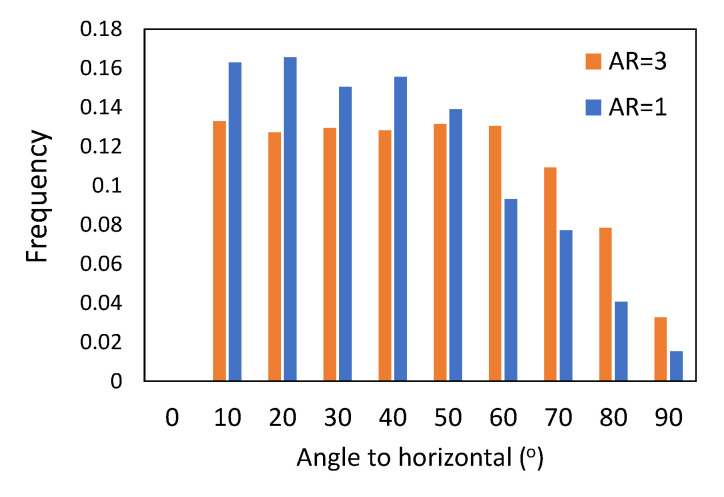
Histogram of vertical orientation of AR = 1 (25%) and AR = 3 (75%) cylinders in initial packing.

**Figure 5:**
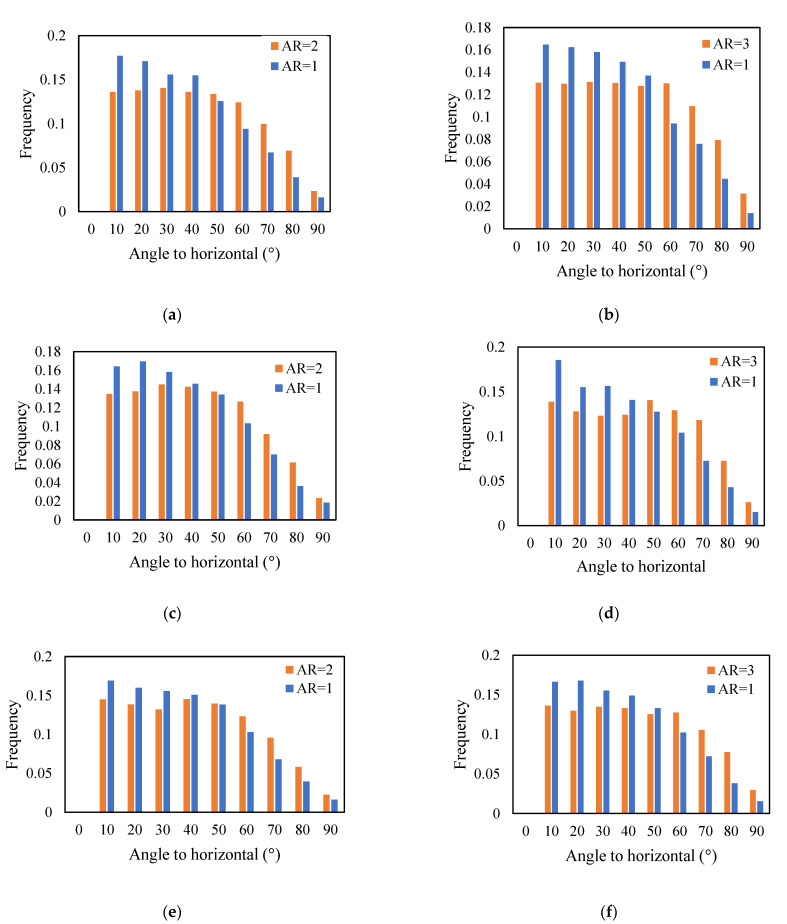
Histogram of vertical orientation of cylinders in final packings of binary mixtures: (**a**) AR = 1 (25%) and AR = 2 (75%), (**b**) AR = 1 (25%) and AR = 3 (75%), (**c**) AR = 1 (50%) and AR = 2 (50%), (**d**) AR = 1 (50%) and AR = 3 (50%), (**e**) AR = 1 (75%) and AR = 2 (25%) and (**f**) AR = 1 (75%) and AR = 3 (25%).

**Figure 6:**
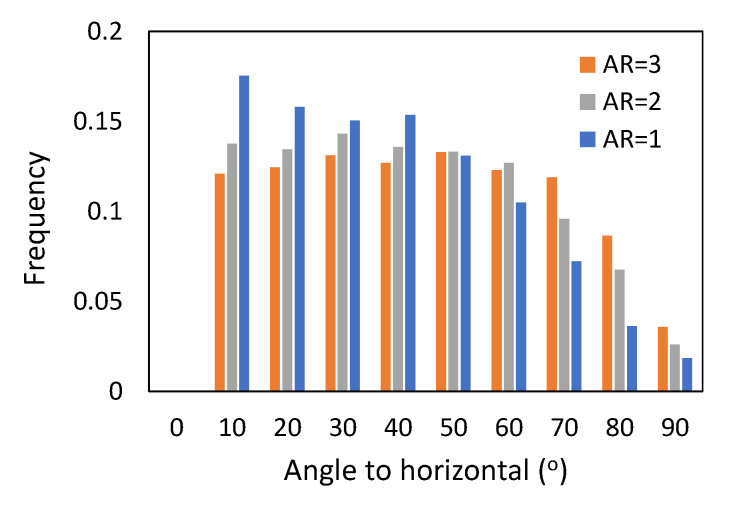
Histogram of vertical orientation of monodisperse cylinders in final packings.

The orientation of cylindrical particles is demonstrated in Figure 4, Figure 5 and Figure 6 in the form of a histogram of the angle between the horizontal plane and the particle axes. Here, an angle of 0° corresponds to the horizontally oriented particle and an angle of 90° to the vertically oriented one. Initially, during the formation of packing by pouring, the particles are randomly oriented, as confirmed in Figure 4. After the formation of stable packed beds, the particles are still randomly oriented, as shown in Figure 5. From Figure 5, we can observe that particles with low aspect ratios, AR = 1, are mainly oriented horizontally, and particles with higher aspect ratios, AR = 2 and AR = 3, are more randomly oriented with angles to the horizontal plane from 10° to 70°, and only 2%–3% of particles are oriented in the vertical direction. Similar trends are observed in Figure 6 for packings of monodisperse cylinders. This can be explained by the fact that the packed beds in our simulations were formed following the poured packing conditions, and the particles falling down from the top of the container tend to orient in the most stable position upon contact with the already formed bed.

### 3.2. Planar Packing Fraction

**Figure 7:**
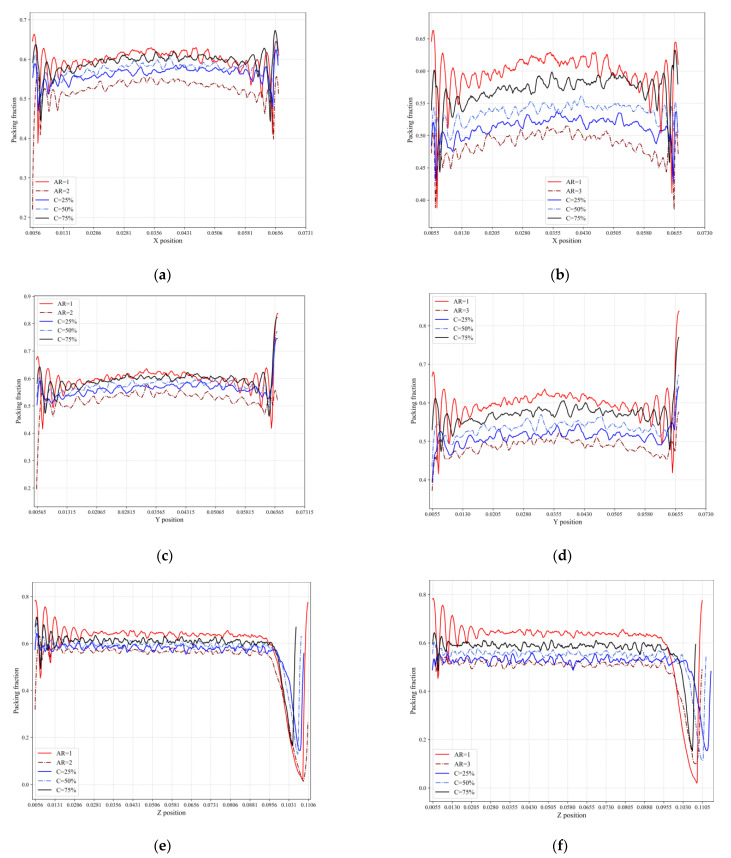
Packing fraction along x,y,z direction for (**a**,**c**,**e**) binary mixtures of AR = 1 and AR = 2, monodisperse AR = 1 and monodisperse AR = 2, and (**b**,**d**,**f**) binary mixtures of AR = 1 and AR = 3, monodisperse AR = 1 and monodisperse AR = 3.

A weak wall effect can be observed from the analysis of the packing fraction variation plots for packings of cylindrical particles (Figure 7). Two prominent peaks can be seen close to the side walls for packings in the *x* and *y* directions, indicating the poor mating of the curved surfaces of the particles against the flat surface of the wall. Significant oscillations in bed porosity near the wall were also reported by Zhang et al. [40] for the packing of equilateral cylindrical particles. The packing fraction in the *z* direction quickly decreases at the end, demonstrating that the particles at the top of the container are not evenly distributed. A similar trend in the axial distribution of local porosity of polydisperse cylindrical particle packed-bed was described by Zhang et al. [41]. The effect of the aspect ratio of cylindrical particles on packing microstructure can be demonstrated by comparing patterns of planar packing fraction distributions for mixtures of different compositions. The closer the aspect ratios of cylinders comprising the binary mixture to each other, the less the packing fraction distributions of mixtures of different composition differ from each other.

### 3.3. Local Packing Structure Analysis

**Figure 8:**
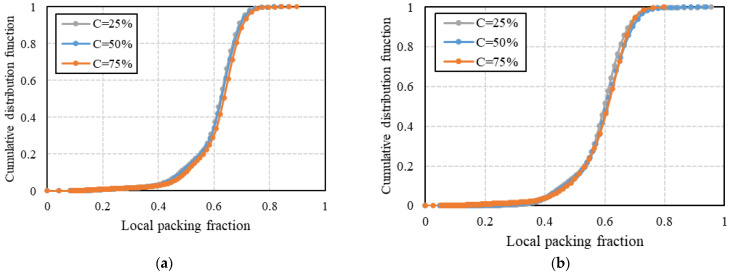
Cumulative distributions of local packing fractions of (**a**) binary mixtures of AR = 1 and AR = 2 and (**b**) binary mixtures of AR = 1 and AR = 3.

**Figure 9:**
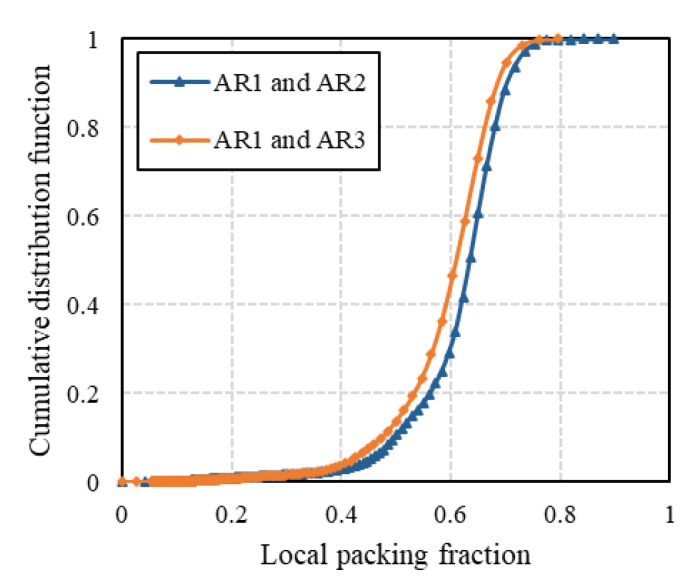
Effect of aspect ratio of cylindrical particles on distribution of local packing fractions.

The cumulative distribution functions (CDFs) of the local packing fraction are shown in Figure 8. The local packing fractions were calculated using the results of Voronoi tessellation as a reciprocal of the reduced Voronoi cell volume. It can be seen that the cumulative distributions of local packing fractions for the mixtures of different compositions show similar patterns. The effect of the aspect ratio of cylindrical particles on the distribution of local packing fractions is illustrated in Figure 9. The binary mixture composed of more elongated particles (AR3) shows a slightly more uniform distribution of local packing fractions with a smaller median value corresponding to the looser packing in comparison with the mixture of AR1 and AR2. 

**Figure 10:**
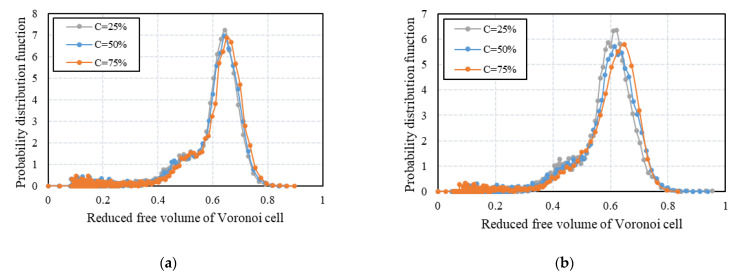
The probability distribution function of the reduced free volume of Voronoi cells of (**a**) binary mixtures AR = 1 and AR = 2, (**b**) binary mixtures AR = 1 and AR = 3.

The probability distribution functions (PDFs) of the reduced free volume of Voronoi cells are represented in Figure 10. These results confirm the conclusion that the mixture of two cylindrical particles with larger differences in aspect ratios results in the loose packings with wider distributions of the reduced free volume of Voronoi cells and the PDFs shift to the larger cells with an increase in the volume fraction of elongated particles.

## 4. Conclusions

This study has focused on the microstructural analysis of poured packings of binary cylindrical particle mixtures with the same volume but different aspect ratios and volume fractions. The particles of approximately cylindrical shape were generated using superquadrics. By applying the DEM approach, binary mixtures of the cylindrical particles were simulated, and their packing microstructure was thoroughly analyzed via voxelization and Voronoi tessellation. 

The simulation results demonstrate that the particles with the larger aspect ratio in the binary mixture tend to orient randomly, and the particles with the smaller aspect ratio are preferentially horizontally oriented. The planar packing fraction curves show that 75% of elongated particles mixed with the 25% cylinders with AR = 1 demonstrate the lowest packing fraction. The cumulative distribution functions of local packing fractions are more uniform and have smaller median values corresponding to the looser packing for mixtures with more elongated cylindrical particles. The probability distribution functions of the reduced free volume of Voronoi cells become wider and shift to larger cell volumes with the increase of particle aspect ratio and volume fraction of cylinders with high aspect ratio. The microstructural characterization of the packing can be used in understanding the local packing structure. The limitation of the present research is that the cylindrical particles generated using superquadrics are geometrically symmetrical and convex with smooth boundaries. The packing structure of such particles could differ from the packing structure of the cylinders with planar faces. Therefore, the present research will be extended in the future by considering the packing of polydisperse cylinders with planar faces.

## Figures and Tables

**Table 1:** Dimension and parameters of cylindrical particles generated using SQ approach.

Sample	Cylinder 1	Cylinder 2	Cylinder 3
	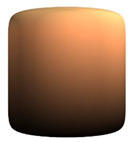	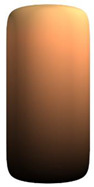	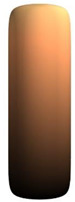
*d*, mm	3	2.381	2.080
AR (h/d)	1	2	3
*h*, mm	3	4.752	6.240
*V*, mm^3^	21.206	21.206	21.206
*a*, mm	1.5	1.191	1.04
*b*, mm	1.5	1.191	1.04
*c*, mm	1.5	2.381	3.12
$n_{1}$	10	10	10
$n_{2}$	2	2	2

**Table 2:** Mechanical properties of stainless-steel particles and parameters of DEM simulations [37].

	Properties	Value
Container size	width × thick × height, m	0.064 × 0.064 × 0.13
Mechanical properties	Young’s modulus, [Pa]	2.2 × 10^8^
	Poisson ratio	0.3
	Restitution coefficient	0.64
	Friction coefficient	0.6
DEM parameters	Time-step, Δt [s]	10^−5^
	Gravity, g [m/s^2^]	9.81
Particles physical properties	Density, [kg/cm^3^]	7980

**Table 3:** Composition of binary mixtures of cylindrical particles.

	Volume Fraction of AR = 1	AR = 1	AR = 2	AR = 3
Binary mixture of AR = 1 and AR = 2	C = 25%	3000	9000	0
	C = 50%	6000	6000	0
	C = 75%	9000	3000	0
Binary mixture of AR = 1 and AR = 3	C = 25%	3000	0	9000
	C = 50%	6000	0	6000
	C = 75%	9000	0	3000

## Data Availability

Not applicable.
